# Supplementary material for: Investigation into the effects of antioxidant-rich extract of Tamarindus indica leaf on antioxidant enzyme activities, oxidative stress and gene expression profiles in HepG2 cells
Source: PeerJ. 2015 Oct 1;3:e1292. doi: 10.7717/peerj.1292 (PMC4636403; doi:10.7717/peerj.1292)
Supplement: Figure S1 [file peerj-03-1292-s001.pdf]

Supplemental Information

Figure S1

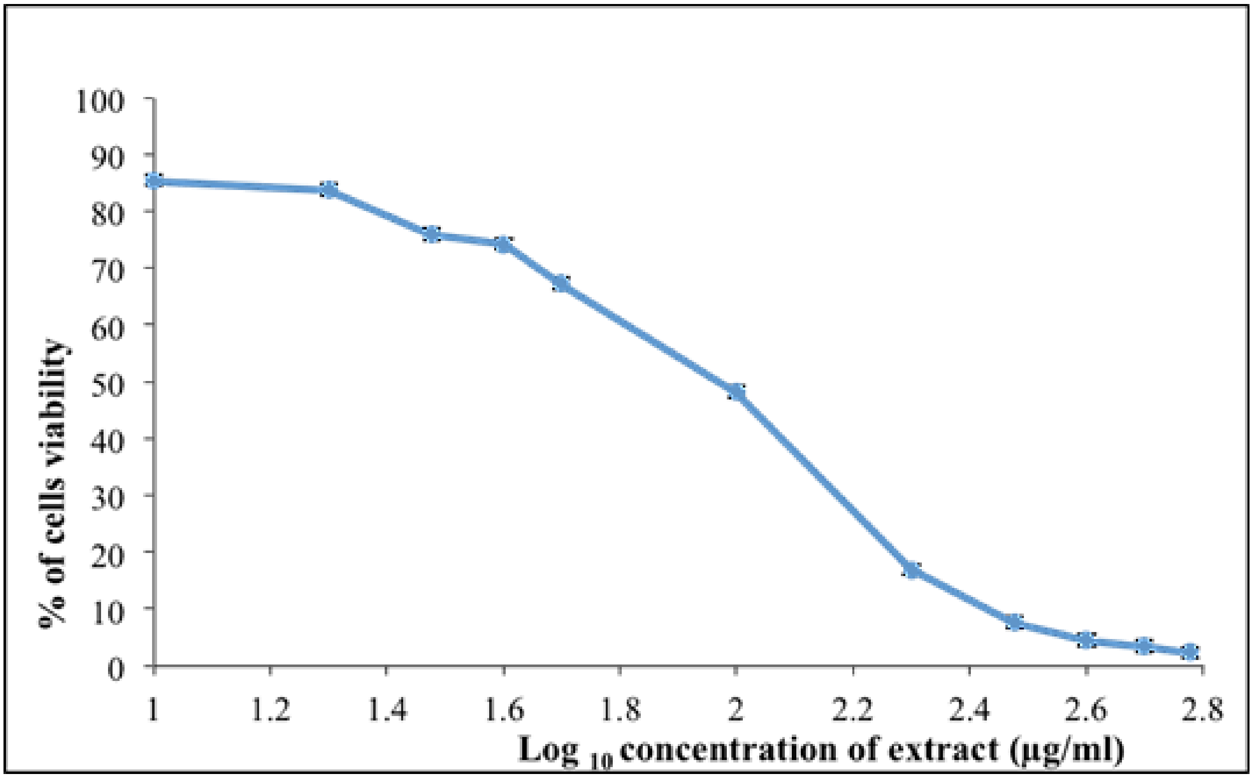

Figure S1: A log plot of concentrations of the methanol leaf extract of *T. indica* (µg/ml) versus percentage (%) of HepG2 cells viability. From the log_{10} plot, the IC_{50} was found to be 93.33 µg/ml and IC_{20} was 24.55µg/ml.
