# Supplementary material for: Investigation into the effects of antioxidant-rich extract of Tamarindus indica leaf on antioxidant enzyme activities, oxidative stress and gene expression profiles in HepG2 cells
Source: PeerJ. 2015 Oct 1;3:e1292. doi: 10.7717/peerj.1292 (PMC4636403; doi:10.7717/peerj.1292)
Supplement: Table S1 [file peerj-03-1292-s002.docx]

Supplemental Information

Table S1: Primer sequences for the selected genes used for validation of the microarray data using real-time RT–PCR (qRT-PCR)

| Gene | Primer sequence  (5’ – 3’) | PCR product size (bp) |
| --- | --- | --- |
| *Fibrinogen alpha chain, FGA* (NM_000508) | Forward:  5’- TCGCCCTGTCAGAGACTGTGATGA – 3’  Reverse:  5’- AGCCATCCTCCCAAACTGGTCTCT – 3’ | 141 |
| *Fibrinogen gamma chain, FGG* (NM_021870) | Forward:  5’- TGGCACAACAGAATTTTGGCTGGGA- 3’  Reverse:  5’ – TGCAGTACTGGTTCTGCCATTCCA – 3’ | 118 |
| *Cytochrome P450, family 24, subfamily A, polypeptide 1,* *CYP24A1* (NM_000782) | Forward:  5’ – ACCCAAAGGAACAGTGCTCATGC – 3’  Reverse:  5’ – ACGCCAAATGGAAGATGCGCAAA – 3’ | 144 |
| *Liver expressed antimicrobial peptide 2,* *LEAP2* (NM_052971) | Forward:  5’ – GGGCCAGATAGATGGCTCCCCA – 3’  Reverse:  5’ – TCCCGGCAGGAGGCTCCAAT – 3’ | 117 |
| *Amphiregulin, AREG* (NM_001657) | Forward:  5’ – CCCCAAGCCTTCGAGAGCGG – 3’  Reverse:  5’ – CGGTCTCTGGGGCAACTCGG – 3’ | 109 |
| *Serpin peptidase inhibitor, clade E, member 1,* *SERPINE1* (NM_000602) | Forward:  5’ – CCTCTGAGAACTTCAGGATGCAG – 3’  Reverse:  5’ – CCTGCTGAAACACCCTCACC – 3’ | 153 |
| *Interferon gamma receptor 1,* *IFNGR1* (NM_000416) | Forward:  5’ – TCGGTAGCAGCATGGCTCTC – 3’  Reverse:  5’ – TTAGTTGGTGTAGGCACTGAGGA – 3’ | 112 |
| *Annexin A3*, *ANXA3* (NM_005139) | Forward:  5’ – ATCAGGTGGAGTCGAGAGGC – 3’  Reverse:  5’ - ACACTAATCCAAAGCGCGG – 3’ | 168 |
| *Mevalonate kinase,* *MVK* (NM_000431) | Forward:  5’- TTCCCAGGAGCCATGTTGTC – 3’  Reverse:  5’ – TACAGCCAGTGCTACCTTGC – 3’ | 105 |
| *24-dehydrocholesterol reductase,* *DHCR24* (NM_014762) | Forward:  5’ - ATGCACTCCGTCCGAAAACT – 3’  Reverse:  5’ - TCGAAACGCAGCTTGACGTA – 3’ | 126 |
| *Aldehyde dehydrogenase 6 family, member A1, ALDH6A1* (NM_005589) | Forward:  5’ - AGGTCTTGCTCCGCTATCAAC – 3’  Reverse:  5’ - GGCATGCTCAACCACCTGAAG – 3’ | 137 |
| *Alcohol dehydrogenase 6, ADH6* (NM_001102470) | Forward:  5’ - GGCCAGTTGTTCTTCTCAGGA – 3’  Reverse:  5’ - ACAGCGGATACATTTTCCAGT – 3’ | 198 |
