# Supplementary material for: Investigation into the effects of antioxidant-rich extract of Tamarindus indica leaf on antioxidant enzyme activities, oxidative stress and gene expression profiles in HepG2 cells
Source: PeerJ. 2015 Oct 1;3:e1292. doi: 10.7717/peerj.1292 (PMC4636403; doi:10.7717/peerj.1292)
Supplement: Table S2 [file peerj-03-1292-s003.docx]

Supplemental Information

Table S2

List of up-regulated genes in HepG2 cells treated with the methanol leaf extract of *T. indica* with fold changes ≥ 1.5 (p < 0.05)

| **Gene Assignment** | **Gene Symbol** | **p-value** | **Fold-Change** |
| --- | --- | --- | --- |
| NM_001040 - SHBG - sex hormone-binding globulin | SHBG | 0.0354 | 4.11 |
| NM_006841 - SLC38A3 - solute carrier family 38, member 3 | SLC38A3 | 0.0385 | 3.69 |
| NM_000371 - TTR - transthyretin | TTR | 0.0276 | 3.40 |
| NM_004590 - CCL16 - chemokine (C-C motif) ligand 16 | CCL16 | 0.0327 | 3.38 |
| NM_001010845 - ACSM2A - acyl-CoA synthetase medium-chain family member 2A | ACSM2A | 0.0058 | 3.21 |
| NM_000508 - FGA - fibrinogen alpha chain | FGA | 0.0222 | 3.03 |
| NM_001007253 - ERV3 - endogenous retroviral sequence 3 | ERV3 | 0.0343 | 2.87 |
| NM_006714 - SMPDL3A - sphingomyelin phosphodiesterase, acid-like 3A | SMPDL3A | 0.0124 | 2.79 |
| NM_000602 - SERPINE1 - serpin peptidase inhibitor, clade E | SERPINE1 | 0.0057 | 2.75 |
| NM_001713 - BHMT - betaine--homocysteine S-methyltransferase | BHMT | 0.0371 | 2.65 |
| NM_182617 - ACSM2B - acyl-CoA synthetase medium-chain family member 2B | ACSM2B | 0.0173 | 2.65 |
| NM_003273 - TM7SF2 - transmembrane 7 superfamily member 2 | TM7SF2 | 0.0083 | 2.60 |
| NM_018285 - IMP3 - IMP3, U3 small nucleolar ribonucleoprotein, homolog (yeast) | IMP3 | 0.0470 | 2.52 |
| NM_000151 - G6PC - glucose-6-phosphatase, catalytic subunit | G6PC | 0.0268 | 2.50 |
| NM_138393 - REEP6 - receptor accessory protein 6 | REEP6 | 0.0051 | 2.47 |
| NM_133491 - SAT2 - spermidine/spermine N1-acetyltransferase family member 2 | SAT2 | 0.0412 | 2.41 |
| NM_021870 - FGG - fibrinogen gamma chain | FGG | 0.0158 | 2.39 |
| NM_174936 - PCSK9 - proprotein convertase subtilisin/kexin type 9 | PCSK9 | 0.0408 | 2.39 |
| NM_025225 - PNPLA3 - patatin-like phospholipase domain containing 3 | PNPLA3 | 0.0123 | 2.31 |
| NM_030792 - GDPD5 - glycerophosphodiester phosphodiesterase domain | GDPD5 | 0.0043 | 2.18 |
| NM_005063 - SCD - stearoyl-CoA desaturase (delta-9-desaturase) | SCD | 0.0263 | 2.17 |
| NM_001080393 - GXYLT2 - glucoside xylosyltransferase 2 | GXYLT2 | 0.0133 | 2.14 |
| NM_000488 - SERPINC1 - serpin peptidase inhibitor, clade C (antithrombin) | SERPINC1 | 0.0422 | 2.14 |
| NM_001025195 - CES1 - carboxylesterase 1 | CES1 | 0.0446 | 2.13 |
| NM_000120 - EPHX1 - epoxide hydrolase 1, microsomal (xenobiotic) | EPHX1 | 0.0256 | 2.12 |
| NM_152419 - HGSNAT - heparan-alpha-glucosaminide N-acetyltransferase | HGSNAT | 0.0436 | 2.01 |
| NM_030943 - AMN - amnionless homolog | AMN | 0.0211 | 1.98 |
| NM_001343 - DAB2 - disabled homolog 2, mitogen-responsive phosphoprotein | DAB2 | 0.0114 | 1.98 |
| NM_002340 - LSS - lanosterol synthase (2,3-oxidosqualene-lanosterol cyclase) | LSS | 0.0203 | 1.97 |
| NM_052971 - LEAP2 - liver expressed antimicrobial peptide 2 | LEAP2 | 0.0347 | 1.96 |
| NM_144676 - TMED6 - transmembrane emp24 protein transport domain containing 6 | TMED6 | 0.0031 | 1.95 |
| NM_015974 - CRYL1 - crystallin, lambda 1 | CRYL1 | 0.0258 | 1.95 |
| NM_006610 - MASP2 - mannan-binding lectin serine peptidase 2 | MASP2 | 0.0308 | 1.92 |
| NM_014762 - DHCR24 - 24-dehydrocholesterol reductase | DHCR24 | 0.0106 | 1.91 |
| NM_080669 - SLC46A1 - solute carrier family 46 (folate transporter), member 1 | SLC46A1 | 0.0044 | 1.90 |
| NM_177550 - SLC13A5 - solute carrier family 13, alpha 5 | SLC13A5 | 0.0118 | 1.89 |
| NM_000898 - MAOB - monoamine oxidase B | MAOB | 0.0245 | 1.88 |
| NM_022746 - MOSC1 - MOCO sulphurase C-terminal domain containing 1 | MOSC1 | 0.0126 | 1.86 |
| NM_005589 - ALDH6A1 - aldehyde dehydrogenase 6 family, member A1 | ALDH6A1 | 0.0418 | 1.86 |
| NM_198563 - TMEM110 - transmembrane protein 110 | TMEM110 | 0.0020 | 1.85 |
| NM_152391 - PQLC3 - PQ loop repeat containing 3 | PQLC3 | 0.0322 | 1.85 |
| NM_000893 - KNG1 - kininogen 1 | KNG1 | 0.0342 | 1.84 |
| NM_014652 - IPO13 - importin 13 | IPO13 | 0.0468 | 1.83 |
| NM_001964 - EGR1 - early growth response 1 | EGR1 | 0.0143 | 1.83 |
| NM_000850 - GSTM4 - glutathione S-transferase mu 4 | GSTM4 | 0.0207 | 1.83 |
| NM_005768 - LPCAT3 - lysophosphatidylcholine acyltransferase 3 | LPCAT3 | 0.0342 | 1.83 |
| NM_019101 - APOM - apolipoprotein M | APOM | 0.0254 | 1.80 |
| NM_001077238 - SPPL2B - signal peptide peptidase-like 2B | SPPL2B | 0.0397 | 1.79 |
| NM_001135099 - TMPRSS2 - transmembrane protease, serine 2 | TMPRSS2 | 0.0250 | 1.78 |
| NM_012340 - NFATC2 - nuclear factor of activated T-cells, cytoplasmic, calcine | NFATC2 | 0.0050 | 1.77 |
| NM_001045 - SLC6A4 - solute carrier family 6 | SLC6A4 | 0.0025 | 1.76 |
| NM_014399 - TSPAN13 - tetraspanin 13 | TSPAN13 | 0.0457 | 1.74 |
| NM_152321 - ERP27 - endoplasmic reticulum protein 27 | ERP27 | 0.0082 | 1.74 |
| NM_000431 - MVK - mevalonate kinase | MVK | 0.0157 | 1.72 |
| NM_005116 - SLC23A2 - solute carrier family 23 | SLC23A2 | 0.0290 | 1.71 |
| NM_020062 - SLC2A4RG - SLC2A4 regulator | SLC2A4RG | 0.0168 | 1.70 |
| NM_001145073 - USP27X - ubiquitin specific peptidase 27, X-linked | USP27X | 0.0078 | 1.70 |
| NM_013391 - DMGDH - dimethylglycine dehydrogenase | DMGDH | 0.0467 | 1.69 |
| NM_145230 - ATP6V0E2 - ATPase, H+ transporting V0 subunit e2 | ATP6V0E2 | 0.0049 | 1.69 |
| NM_012079 - DGAT1 - diacylglycerol O-acyltransferase homolog 1 | DGAT1 | 0.0469 | 1.68 |
| NM_004901 - ENTPD4 - ectonucleoside triphosphate diphosphohydrolase 4 | ENTPD4 | 0.0335 | 1.68 |
| NM_000696 - ALDH9A1 - aldehyde dehydrogenase 9 family, member A1 | ALDH9A1 | 0.0316 | 1.67 |
| NM_017567 - NAGK - N-acetylglucosamine kinase | NAGK | 0.0436 | 1.67 |
| NM_007109 - TCF19 - transcription factor 19 | TCF19 | 0.0163 | 1.67 |
| NR_024532 - ALG2 - asparagine-linked glycosylation 2 | ALG2 | 0.0367 | 1.66 |
| NM_001102470 - ADH6 - alcohol dehydrogenase 6 (class V) | ADH6 | 0.0484 | 1.65 |
| NM_004147 - DRG1 - developmentally regulated GTP binding protein 1 | DRG1 | 0.0340 | 1.65 |
| NM_015036 - ENDOD1 - endonuclease domain containing 1 | ENDOD1 | 0.0090 | 1.64 |
| NM_001098721 - GNG4 - guanine nucleotide binding protein (G protein), gamma 4 | GNG4 | 0.0483 | 1.64 |
| NM_032508 - TMEM185A - transmembrane protein 185A | TMEM185A | 0.0406 | 1.64 |
| NM_006214 - PHYH - phytanoyl-CoA 2-hydroxylase | PHYH | 0.0231 | 1.63 |
| NM_033285 - TP53INP1 - tumor protein p53 inducible nuclear protein 1 | TP53INP1 | 0.0096 | 1.63 |
| NM_004107 - FCGRT - Fc fragment of IgG, receptor, transporter, alpha | FCGRT | 0.0304 | 1.62 |
| NM_001040152 - PEG10 - paternally expressed 10 | PEG10 | 0.0334 | 1.59 |
| NR_024484 - LOC400657 - hypothetical LOC400657 | LOC400657 | 0.0082 | 1.59 |
| NM_012106 - ARL2BP - ADP-ribosylation factor-like 2 binding protein | ARL2BP | 0.0125 | 1.59 |
| NM_000277 - PAH - phenylalanine hydroxylase | PAH | 0.0144 | 1.58 |
| NM_002462 - MX1 - myxovirus (influenza virus) resistance 1 | MX1 | 0.0118 | 1.58 |
| NM_000185 - SERPIND1 - serpin peptidase inhibitor, clade D (heparin cofactor) | SERPIND1 | 0.0015 | 1.58 |
| NM_019029 - CPVL - carboxypeptidase, vitellogenic-like | CPVL | 0.0027 | 1.57 |
| NM_018672 - ABCA5 - ATP-binding cassette, sub-family A (ABC1), member 5 | ABCA5 | 0.0419 | 1.57 |
| NM_139015 - SPPL3 - signal peptide peptidase 3 | SPPL3 | 0.0233 | 1.56 |
| NM_014015 - DEXI - Dexi homolog (mouse) | DEXI | 0.0494 | 1.56 |
| NM_212472 - PRKAR1A - protein kinase, cAMP-dependent, regulatory, type I, alpha | PRKAR1A | 0.0406 | 1.55 |
| NM_018218 - USP40 - ubiquitin specific peptidase 40 | USP40 | 0.0310 | 1.55 |
| NM_015107 - PHF8 - PHD finger protein 8 | PHF8 | 0.0260 | 1.55 |
| NM_001105663 - NUDT7 - nudix (nucleoside diphosphate linked moiety X) | NUDT7 | 0.0276 | 1.54 |
| NM_001102402 - PCTP - phosphatidylcholine transfer protein | PCTP | 0.0140 | 1.54 |
| NM_003851 - CREG1 - cellular repressor of E1A-stimulated genes 1 | CREG1 | 0.0099 | 1.54 |
| NM_020648 - TWSG1 - twisted gastrulation homolog 1 (Drosophila) | TWSG1 | 0.0081 | 1.54 |
| NM_000775 - CYP2J2 - cytochrome P450, family 2, subfamily J, polypeptide 2 | CYP2J2 | 0.0236 | 1.54 |
| NM_020179 - C11orf75 - chromosome 11 open reading frame 75 | C11orf75 | 0.0398 | 1.52 |
| NM_001710 - CFB - complement factor B | CFB | 0.0069 | 1.52 |
| NM_007054 - KIF3A - kinesin family member 3A | KIF3A | 0.0461 | 1.52 |
| NM_052944 - SLC5A11 - solute carrier family 5 (sodium/glucose cotransporter) | SLC5A11 | 0.0139 | 1.51 |
| NM_000416 - IFNGR1 - interferon gamma receptor 1 | IFNGR1 | 0.0341 | 1.51 |
| NM_032562 - PLA2G12B - phospholipase A2, group XIIB | PLA2G12B | 0.0324 | 1.50 |
| NM_138341 - TMEM116 - transmembrane protein 116 | TMEM116 | 0.0201 | 1.49 |
| NM_014369 - PTPN18 - protein tyrosine phosphatase, non-receptor type 18 | PTPN18 | 0.0307 | 1.49 |
| NM_153345 - TMEM139 - transmembrane protein 139 | TMEM139 | 0.0386 | 1.48 |
| NM_001080419 - UNK - unkempt homolog (Drosophila) | UNK | 0.0466 | 1.48 |
| NM_016930 - STX18 - syntaxin 18 | STX18 | 0.0144 | 1.48 |
| NM_018571 - STRADB - STE20-related kinase adaptor beta | STRADB | 0.0016 | 1.48 |
| NM_007024 - TMEM115 - transmembrane protein 115 | TMEM115 | 0.0155 | 1.48 |
| NM_001128615 - ARHGEF3 - Rho guanine nucleotide exchange factor (GEF) 3 | ARHGEF3 | 0.0179 | 1.47 |
| NM_032436 - ZNF828 - zinc finger protein 828 | ZNF828 | 0.0473 | 1.47 |
| NM_014585 - SLC40A1 - solute carrier family 40 (iron-regulated transporter) | SLC40A1 | 0.0103 | 1.46 |
| NM_022465 - IKZF4 - IKAROS family zinc finger 4 | IKZF4 | 0.0433 | 1.46 |
| NM_147128 - ZNRF2 - zinc and ring finger 2 | ZNRF2 | 0.0195 | 1.45 |
| NM_002615 - SERPINF1 - serpin peptidase inhibitor, clade F | SERPINF1 | 0.0070 | 1.45 |

List of down-regulated genes in HepG2 cells treated with the methanol leaf extract of *T. indica* with fold changes ≥ 1.5 (p < 0.05)

| **Gene Assignment** | **Gene Symbol** | **p-value** | Fold-Change |
| --- | --- | --- | --- |
| NM_031958 - KRTAP3-1 - keratin associated protein 3 | KRTAP3-1 | 0.0439 | -5.53 |
| NM_002167 - ID3 - inhibitor of DNA binding 3 | ID3 | 0.0436 | -4.29 |
| NM_000782 - CYP24A1 - cytochrome P450, family 24, subfamily A, polypeptide1 | CYP24A1 | 0.0015 | -4.19 |
| NM_001701 - BAAT - bile acid CoA: amino acid N-acyltransferase | BAAT | 0.0091 | -3.45 |
| NM_005139 - ANXA3 - annexin A3 | ANXA3 | 0.0412 | -3.32 |
| NM_014391 - ANKRD1 - ankyrin repeat domain 1 (cardiac muscle) | ANKRD1 | 0.0168 | -3.25 |
| NM_005100 - AKAP12 - A kinase (PRKA) anchor protein 12 | AKAP12 | 0.0391 | -3.13 |
| NM_002960 - S100A3 - S100 calcium binding protein A3 | S100A3 | 0.0274 | -3.05 |
| ENST00000383686 - LOC151760 - hypothetical LOC151760 | LOC151760 | 0.0068 | -2.82 |
| NM_033049 - MUC13 - mucin 13, cell surface associated | MUC13 | 0.0178 | -2.69 |
| NR_003033 - SNORD5 - small nucleolar RNA, C/D box 5 | SNORD5 | 0.0425 | -2.59 |
| NM_153608 - ZNF114 - zinc finger protein 114 | ZNF114 | 0.0200 | -2.57 |
| NM_003641 - IFITM1 - interferon induced transmembrane protein 1 | IFITM1 | 0.0313 | -2.46 |
| NM_006650 - CPLX2 - complexin 2 | CPLX2 | 0.0072 | -2.40 |
| NM_001562 - IL18 - interleukin 18 (interferon-gamma-inducing factor) - 11q22. | IL18 | 0.0405 | -2.39 |
| NM_001554 - CYR61 - cysteine-rich, angiogenic inducer, 61 - 1p31-p22 - 3491 | CYR61 | 0.0026 | -2.35 |
| NM_001122 - PLIN2 - perilipin | PLIN2 | 0.0086 | -2.33 |
| NM_001145829 - TNNI2 - troponin I type 2 | TNNI2 | 0.0300 | -2.32 |
| NM_001975 - ENO2 - enolase 2 | ENO2 | 0.0273 | -2.32 |
| NM_001128127 - GK - glycerol kinase | GK | 0.0330 | -2.27 |
| NM_004760 - STK17A - serine/threonine kinase | STK17A | 0.0313 | -2.22 |
| NM_002577 - PAK2 - p21 protein (Cdc42/Rac)-activated kinase 2 | PAK2 | 0.0320 | -2.16 |
| NM_003670 - BHLHE40 - basic helix-loop-helix family, member e40 | BHLHE40 | 0.0106 | -2.15 |
| NM_001657 - AREG - amphiregulin | AREG | 0.0230 | -2.13 |
| NM_015440 - MTHFD1L - methylenetetrahydrofolate | MTHFD1L | 0.0118 | -2.08 |
| NM_004900 - APOBEC3B - apolipoprotein B mRNA editing enzyme | APOBEC3B | 0.0349 | -2.06 |
| NM_173540 - FUT11 - fucosyltransferase 11 | FUT11 | 0.0021 | -2.03 |
| NM_153449 - SLC2A14 - solute carrier family 2 | SLC2A14 | 0.0463 | -2.02 |
| NR_024151 - HSPA7 - heat shock 70kDa protein 7 | HSPA7 | 0.0368 | -2.01 |
| BC063894 - DENND2C - DENN/MADD domain containing 2C | DENND2C | 0.0001 | -1.97 |
| NM_015440 - MTHFD1L - methylenetetrahydrofolate dehydrogenase | MTHFD1L | 0.0167 | -1.94 |
| NM_001013732 - C6orf138 - chromosome 6 open reading frame 138 | C6orf138 | 0.0037 | -1.93 |
| NM_001143942 - RBM24 - RNA binding motif protein 24 | RBM24 | 0.0392 | -1.91 |
| NM_033119 - NKD1 - naked cuticle homolog 1 | NKD1 | 0.0472 | -1.91 |
| NM_004099 - STOM - stomatin - 9q34.1 - 2040 -/ NM_198194 - STOM - stomati | STOM | 0.0126 | -1.90 |
| NM_015440 - MTHFD1L - methylenetetrahydrofolate dehydrogenase | MTHFD1L | 0.0349 | -1.89 |
| NM_000189 - HK2 - hexokinase | HK2 | 0.0272 | -1.88 |
| NM_024580 - EFTUD1 - elongation factor Tu GTP binding domain containing 1 | EFTUD1 | 0.0138 | -1.85 |
| NM_002498 - NEK3 - NIMA (never in mitosis gene a)-related kinase 3 | NEK3 | 0.0218 | -1.85 |
| NM_017763 - RNF43 - ring finger protein 43 | RNF43 | 0.0329 | -1.84 |
| NM_006187 - OAS3 - 2'-5'-oligoadenylate synthetase 3 | OAS3 | 0.0309 | -1.81 |
| NM_206922 - CRIP3 - cysteine-rich protein 3 | CRIP3 | 0.0447 | -1.78 |
| NM_006295 - VARS - valyl-tRNA | VARS | 0.0279 | -1.76 |
| NM_024913 - C7orf58 - chromosome 7 open reading frame 58 | C7orf58 | 0.0285 | -1.75 |
| NM_017784 - OSBPL10 - oxysterol binding protein-like | OSBPL10 | 0.0084 | -1.73 |
| NR_002824 - HERC2P2 - hect domain and RLD 2 pseudogene 2 | HERC2P2 | 0.0102 | -1.72 |
| NM_006017 - PROM1 - prominin 1 | PROM1 | 0.0445 | -1.72 |
| NM_001008493 - ENAH - enabled homolog | ENAH | 0.0096 | -1.70 |
| NM_012262 - HS2ST1 - heparan sulfate 2-O-sulfotransferase 1 | HS2ST1 | 0.0198 | -1.68 |
| NC_000014 - RPL7AP6 - ribosomal protein L7a pseudogene 6 | RPL7AP6 | 0.0104 | -1.68 |
| NM_019096 - GTPBP2 - GTP binding protein | GTPBP2 | 0.0209 | -1.67 |
| NM_005245 - FAT1 - FAT tumor suppressor homolog 1 | FAT1 | 0.0309 | -1.66 |
| NR_027634 - FAM35B2 - family with sequence similarity 35, member B2 | FAM35B2 | 0.0332 | -1.65 |
| NM_030962 - SBF2 - SET binding factor 2 | SBF2 | 0.0047 | -1.65 |
| NM_001143979 - NDE1 - nudE nuclear distribution gene E homolog 1 | NDE1 | 0.0479 | -1.65 |
| NM_003983 - SLC7A6 - solute carrier family 7 | SLC7A6 | 0.0272 | -1.63 |
| NM_001080415 - SR140 - U2-associated SR140 protein | SR140 | 0.0499 | -1.63 |
| NM_015201 - BOP1 - block of proliferation 1 | BOP1 | 0.0250 | -1.61 |
| NM_001024948 - FNBP1L - formin binding protein 1-like | FNBP1L | 0.0113 | -1.60 |
| NM_016542 - MST4 - serine/threonine protein kinase MST4 | MST4 | 0.0116 | -1.60 |
| NM_000292 - PHKA2 - phosphorylase kinase, alpha 2 (liver) | PHKA2 | 0.0221 | -1.60 |
| NM_018948 - ERRFI1 - ERBB receptor feedback inhibitor 1 - | ERRFI1 | 0.0234 | -1.59 |
| NM_014018 - MRPS28 - mitochondrial ribosomal protein S28 | MRPS28 | 0.0350 | -1.59 |
| NM_001255 - CDC20 - cell division cycle 20 homolog | CDC20 | 0.0201 | -1.59 |
| NM_020375 - C12orf5 - chromosome 12 open reading frame 5 - | C12orf5 | 0.0360 | -1.58 |
| NM_005911 - MAT2A - methionine adenosyltransferase II, alpha | MAT2A | 0.0289 | -1.58 |
| NM_000917 - P4HA1 - prolyl 4-hydroxylase, alpha polypeptide I | P4HA1 | 0.0449 | -1.57 |
| NM_007145 - ZNF146 - zinc finger protein 146 | ZNF146 | 0.0182 | -1.57 |
| NM_001146274 - TCF7L2 - transcription factor 7-like 2 ( | TCF7L2 | 0.0103 | -1.56 |
| NM_005371 - METTL1 - methyltransferase like 1 | METTL1 | 0.0282 | -1.54 |
| NM_032199 - ARID5B - AT rich interactive domain 5B (MRF1-like) | ARID5B | 0.0311 | -1.54 |
| NM_002381 - MATN3 - matrilin 3 | MATN3 | 0.0192 | -1.54 |
| NM_152995 - NFXL1 - nuclear transcription factor, X-box binding-like 1 - | NFXL1 | 0.0164 | -1.54 |
| NM_019613 - WDR45L - WDR45-like | WDR45L | 0.0363 | -1.54 |
| NM_004926 - ZFP36L1 - zinc finger protein 36, C3H type-like 1 | ZFP36L1 | 0.0482 | -1.54 |
| NM_003249 - THOP1 - thimet oligopeptidase 1 - | THOP1 | 0.0449 | -1.53 |
| NM_019852 - METTL3 - methyltransferase like 3 | METTL3 | 0.0066 | -1.53 |
| NM_024907 - FBXO17 - F-box protein 17 | FBXO17 | 0.0202 | -1.52 |
| NM_033010 - PCBP4 - poly(rC) binding protein 4 | PCBP4 | 0.0280 | -1.51 |
| NM_004566 - PFKFB3 - 6-phosphofructo-2-kinase/fructose-2,6-biphosphatase 3 | PFKFB3 | 0.0232 | -1.50 |
| NM_198076 - FAM36A - family with sequence similarity 36, member A | FAM36A | 0.0001 | -1.49 |
| NM_006734 - HIVEP2 - human immunodeficiency virus type I enhancer binding prot | HIVEP2 | 0.0050 | -1.49 |
| NM_182706 - SCRIB - scribbled homolog | SCRIB | 0.0042 | -1.49 |
| NM_000214 - JAG1 - jagged 1 (Alagille syndrome) | JAG1 | 0.0496 | -1.48 |
| NM_000985 - RPL17 - ribosomal protein L17 | RPL17 | 0.0187 | -1.48 |
| AK295122 - ZNF778 - zinc finger protein 778 | ZNF778 | 0.0059 | -1.48 |
| NM_033286 - C15orf23 - chromosome 15 open reading frame 23 | C15orf23 | 0.0434 | -1.48 |
| NM_004415 - DSP - desmoplakin - | DSP | 0.0051 | -1.47 |
| NR_030754 - MIR622 - microRNA 622 | MIR622 | 0.0240 | -1.47 |
| NM_000339 - SLC12A3 - solute carrier family 12 (sodium/chloride transporters), | SLC12A3 | 0.0222 | -1.47 |
| NM_170711 - DAZAP1 - DAZ associated protein 1 | DAZAP1 | 0.0072 | -1.47 |
| AB096683 - FAM72D - family with sequence similarity 72, member D | FAM72D | 0.0407 | -1.47 |
| NM_006074 - TRIM22 - tripartite motif-containing 22 | TRIM22 | 0.0057 | -1.46 |
| NM_003730 - RNASET2 - ribonuclease T2 | RNASET2 | 0.0103 | -1.46 |
| NM_173474 - NTAN1 - N-terminal asparagine amidase | NTAN1 | 0.0381 | -1.45 |
| NM_001142556 - HMMR - hyaluronan-mediated motility receptor | HMMR | 0.0370 | -1.45 |
| NM_005431 - XRCC2 - X-ray repair complementing defective repair in Chinese ham | XRCC2 | 0.0261 | -1.45 |
